# Infants at School

**Published:** 1902-04-05

**Authors:** 


### INFANTS AT SCHOOL.

At the last meeting of the Childhood Society Dr. Arthur Newsholme raised a question which is likely to become one of very great importance when people begin to recognise the true meaning of, and intent of, education. He pleaded that children under five years of age should be excluded from Public Elementary Schools. In urging this he showed that in England and Wales nearly 11 per cent. of the total school children at all ages were under five years old, and that the chief reason for the increased attendance of these children (under the age at which compulsory attendance begins) were the desire of mothers to go out to work, and the corresponding desire on the part of the school authorities to prevent girls in higher standards being kept from school to help in nursing these young children. Educationally he said there was no gain by commencing school work so early; on the contrary, it was injurious to both brain and eyesight. As to brain he compared English conditions with those which prevailed in Scotland, where only 2·2 per cent. of the total of the children on the register are under five years of age. He also pointed out how wasteful of public money the system was, for at a low estimate £900,000 were spent annually in caring for these infants; but his great argument was derived from the fact that such school attendance increases the prevalence of, and the fatality from, infectious diseases. The death rate from the common infectious diseases, such as measles, whooping cough, scarlet fever, and diphtheria, rapidly decreased with every additional year of life, and he held that by delaying the attacks of these diseases until the first five years were over, thousands of lives might be saved. To sum up, he held that the attendance of children under five years of age presents no educational advantage; that it is inimical to the physical and mental health of the children; that it costs nearly a million sterling every year, and that it causes an enormous annual death-roll from preventable disease. This is a heavy indictment which the educationists will, we think, find it difficult to answer.

